# Durvalumab Monotherapy in Complex Advanced Hepatocellular Carcinoma: A Real‐World Study of Patients Ineligible for Combination Immunotherapy

**DOI:** 10.1002/cam4.70642

**Published:** 2025-02-27

**Authors:** Chihiro Miwa, Sadahisa Ogasawara, Takuya Yonemoto, Sae Yumita, Tomomi Okubo, Miyuki Nakagawa, Keisuke Koroki, Masanori Inoue, Naoya Kanogawa, Masato Nakamura, Takayuki Kondo, Shingo Nakamoto, Norio Itokawa, Masanori Atsukawa, Ei Itobayashi, Naoya Kato

**Affiliations:** ^1^ Department of Gastroenterology, Graduate School of Medicine Chiba University Chiba Japan; ^2^ Department of Gastroenterology, Chiba Hokusoh Hospital Nippon Medical School Tokyo Japan; ^3^ Department of Gastroenterology Asahi General Hospital Asahi Japan

**Keywords:** Child–Pugh B, chronic kidney disease, durvalumab, hepatocellular carcinoma, immunotherapy, real‐world study

## Abstract

**Aim:**

Combination immunotherapy is the standard of care for advanced hepatocellular carcinoma (HCC). However, some patients are unsuitable for such treatment. This study investigated the safety and effectiveness of durvalumab monotherapy in a real‐world cohort with advanced HCC who were poor candidates for combination immunotherapy.

**Methods:**

We retrospectively analyzed data from 35 patients with advanced HCC treated with durvalumab monotherapy across three Japanese institutions between January and December 2023. Patients were selected based on their ineligibility for combination immunotherapy or vascular endothelial growth factor inhibiting tyrosine kinase inhibitors (VEGF‐TKIs). Overall survival (OS), progression‐free survival (PFS), objective response rate (ORR), disease control rate (DCR), and adverse events (AEs) were assessed.

**Results:**

The median age was 71 years, with 51.4% classified as Child–Pugh B or C. Notably, 91.4% of patients were ineligible for the IMbrave150 or HIMALAYA trials. Median PFS was 2.7 months (95% CI: 1.84–6.2) and the median OS was not reached. The ORR and DCR were 2.9% and 51.4%, respectively. Grade ≥ 3 treatment‐related AEs (trAEs) occurred in 8.6% of patients, with a discontinuation rate of 11.4% due to AEs. The most common AEs were aspartate aminotransferase (AST) increased (34.3%), hypoalbuminemia (28.6%), and alanine aminotransferase (ALT) increased (25.7%). Immune‐mediated AEs (imAEs) affected 14.3% of the patients. The albumin‐bilirubin (ALBI) scores showed no significant deterioration in patients without progressive disease (PD) over 12 weeks after treatment initiation (*p* = 0.771).

**Conclusions:**

Durvalumab monotherapy demonstrated a favorable safety profile and comparable effectiveness to VEGF‐TKIs in patients with advanced HCC unsuitable for combination immunotherapy, especially for those with Child–Pugh B status.

**Abbreviations**
AE: adverse event
AFP: alpha‐fetoprotein
ALBI: albumin‐bilirubin
ALT: alanine aminotransferase
AST: aspartate aminotransferase
BCLC: Barcelona Clinic Liver Cancer
CR: complete response
CT: computed tomography
CTCAE: Common Terminology Criteria for Adverse Events
CTLA‐4: cytotoxic T‐lymphocyte‐associated protein 4
DCR: disease control rate
HBV: hepatitis B virus
HCC: hepatocellular carcinoma
HCV: hepatitis C virus
ICI: immune checkpoint inhibitor
imAE: immune‐mediated adverse event
MASLD: metabolic‐associated steatotic liver disease
MRI: magnetic resonance imaging
ORR: objective response rate
OS: overall survival
PD: progressive disease
PD‐1: programmed cell death protein 1
PD‐L1: programmed death‐ligand 1
PFS: progression‐free survival
PR: partial response
RECIST: Response Evaluation Criteria in Solid Tumors
SD: stable disease
Tbil: total bilirubin
trAE: treatment‐related adverse event
VEGF‐TKI: vascular endothelial growth factor‐inhibiting tyrosine kinase inhibitors

## 1 Introduction

The treatment landscape for advanced hepatocellular carcinoma (HCC) has evolved significantly. Three pivotal phase III trials (IMbrave150, HIMALAYA, and CHECKMATE9DW) established combination immunotherapies, including atezolizumab plus bevacizumab, durvalumab plus tremelimumab, and nivolumab plus ipilimumab, as preferred first‐line treatments [1, 2, 3, 4, 5, 6]. While early trials of PD‐1/PD‐L1 inhibitor monotherapy showed limited success, durvalumab monotherapy demonstrated efficacy comparable to sorafenib in the HIMALAYA trial, offering an additional first‐line treatment option [7, 8].

The current guidelines recommend combination immunotherapy for patients with Child–Pugh class A [4, 5, 6]. However, the optimal strategy for individuals with impaired liver function (Child–Pugh B) remains unclear. Although VEGF‐TKIs have demonstrated safety and efficacy in this patient population, the available data on combination immunotherapies are limited [9, 10, 11]. While durvalumab plus tremelimumab represents a potential avenue of treatment, it is often restricted by the occurrence of immune‐mediated adverse events [12, 13]. In Japan, guidelines recommend VEGF‐TKIs or durvalumab monotherapy for patients who are not eligible for combination therapy [4]. The HIMALAYA trial demonstrated that durvalumab monotherapy was non‐inferior to sorafenib with a favorable safety profile [2], while nivolumab has shown potential for patients with Child–Pugh B liver function [14]. However, clinical data on the efficacy of durvalumab in combination therapy‐ineligible patients remain limited. This study investigates the safety and efficacy of durvalumab monotherapy in a real‐world Japanese setting, specifically focusing on patients ineligible for combination immunotherapy.

## 2 Patients and Methods

### 2.1 Patients

We retrospectively analyzed data from patients with advanced HCC treated with durvalumab monotherapy between January and December 2023 at three Japanese institutions. Patient selection was determined by ineligibility for two standard treatment approaches of combination immunotherapy and VEGF‐TKI therapy. VEGF‐TKI exclusion criteria encompassed proteinuria, renal impairment, elevated bleeding risks, thromboembolic events, and poor wound healing [15, 16, 17]. For combination immunotherapy, patients were excluded based on IMbrave050 and HIMALAYA trial criteria, with factors including active autoimmune conditions, kidney dysfunction, hemorrhagic risks, and complications such as ascites or hepatic encephalopathy [1, 2]. Data collection ended in February 2024. This study was approved by the Research Ethics Committee of Chiba University (HK202309‐02).

### 2.2 Treatment With Durvalumab Monotherapy

Durvalumab was administered at 1500 mg every 4 weeks. Tumor response was evaluated using response evaluation criteria in solid tumors version 1.1 (RECIST v1.1) criteria via computed tomography (CT) or magnetic resonance imaging (MRI) every 4–8 weeks. Treatment was continued until disease progression or the occurrence of unacceptable AEs.

### 2.3 Clinical Parameters

We collected baseline demographics, AEs, radiological progression dates, and survival data. Radiological evaluations followed RECIST v1.1, and AEs were assessed using the Common Terminology Criteria for Adverse Events version 4.03 (CTCAE v4.03).

### 2.4 Statistical Analysis

OS and PFS were estimated using Kaplan–Meier plots with 95% CIs. Cox proportional hazards regression was used to identify factors affecting durvalumab administration. Changes in ALBI score were analyzed using a mixed‐effects model. Statistical significance was set at *p* < 0.05. Analyses were conducted using R software.

## 3 Results

### 3.1 Patient Background and Characteristics

A total of 35 patients received durvalumab monotherapy, comprising 24 patients from Chiba University Hospital, 6 from Asahi General Hospital, and 5 from Chiba Hokusoh Hospital of Nippon Medical School. Table 1 summarizes the patients' clinical characteristics. The median age was 71 years (range: 54–88), with 28.6% aged ≥ 80 years. Common underlying liver diseases included hepatitis C virus infection (40.0%), alcoholic liver disease (37.1%), and MASLD (34.3%). Macrovascular invasion was present in 22.9% of patients and extrahepatic metastases in 17.1%. While 48.6% were Child–Pugh A, 51.4% were Child–Pugh B or C, with most patients (57.1%) classified as ALBI grade 2b. Severe proteinuria and renal impairment were observed in 25.7% and 14.3% of patients, respectively.

**TABLE 1:** Baseline characteristics in advanced hepatocellular carcinoma patients who received durvalumab monotherapy.

Characteristic	All (*N* = 35)
Age > 80	10 (28.6)
Gender, male	28 (80.0)
HBV positive	3 (8.6)
HCV positive	14 (40.0)
Alcoholic	13 (37.1)
MASH/MASLD (clinically diagnosed)	12 (34.3)
MetALD	3 (8.6)
Child–Pugh class B–C	18 (51.4)
BCLC stage C	12 (34.3)
ALBI grade 2b‐3	24 (68.5)
Macrovascular invasion	8 (22.9)
Extrahepatic spread	6 (17.1)
ECOG‐PS 1–2	2 (5.7)
AFP > 400 ng/mL	6 (17.1)
eGFR < 50	5 (14.3)
UPC > 0.5	9 (25.7)

*Note:* Values are expressed as *n* (%).

Abbreviations: AFP, alpha‐fetoprotein; BCLC, Barcelona Clinic Liver Cancer; ECOG‐PS, Eastern Cooperative Oncology Group Performance Status; HBV, hepatitis B virus; HCV, hepatitis C virus; MASH, metabolic dysfunction associated steatohepatitis; MASLD, metabolic dysfunction‐associated steatotic liver disease; MetALD, MASLD and increased alcohol intake; UPC, urine protein‐to‐creatinine ratio.

### 3.2 Clinical Course in 35 Patients Receiving Durvalumab Monotherapy

**FIGURE 1:**
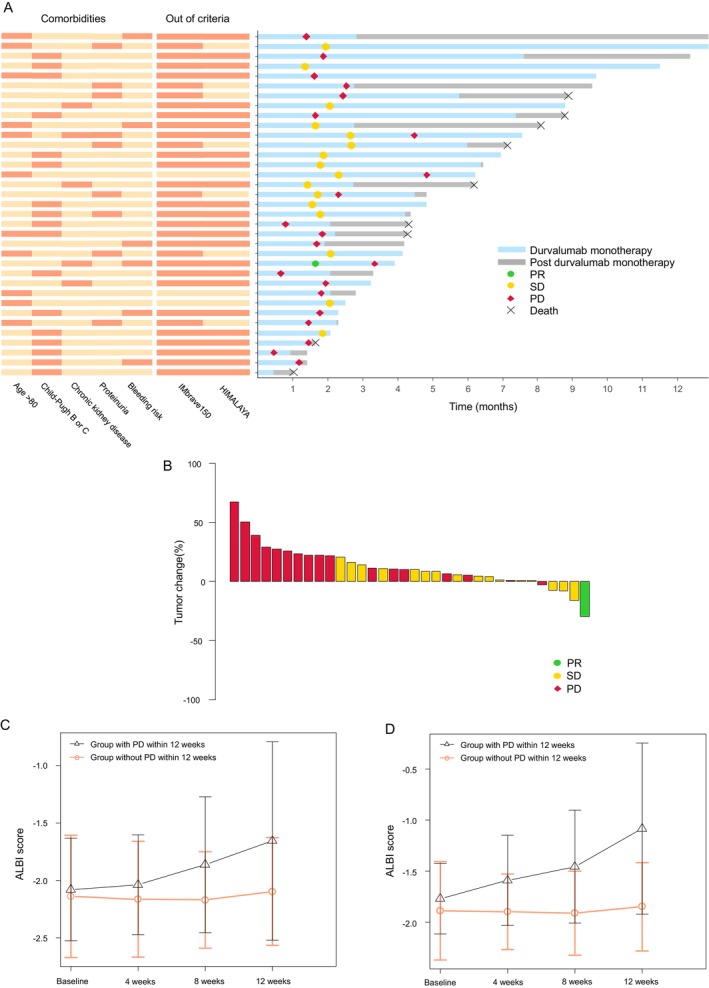
Review of treatment selection criteria, clinical courses, tumor response, and changes in liver function over time. (A) Swimmer plot showing drug administration cases, accompanied by a heatmap of treatment selection reasons and eligibility criteria. The left heatmap (orange) highlights reasons for selecting each treatment, while the right heatmap (orange) indicates cases that did not meet eligibility criteria for the IMbrave150 and HIMALAYA trials. This visualization offers insights into the rationale behind treatment decisions and clinical profiles of the analyzed cases. (B) Waterfall plot illustrating tumor growth rates for individual cases, color‐coded by each patient's best treatment response. (C) Line graph depicting ALBI score changes in patients with and without tumor progression. The mean ALBI scores are shown at 4, 8, and 12 weeks post‐treatment, with the orange line representing patients who experienced tumor progression (defined by increased tumor size) and the black line representing those without progression. Error bars indicate the standard error of the mean. ALBI score, albumin–bilirubin score; OS, overall survival; PD, progressive disease; PFS, progression‐free survival; PR, partial response; SD, stable disease.

Figure 1A illustrates the clinical course of the 35 patients with advanced HCC who received durvalumab monotherapy. The five key factors for durvalumab selection were advanced age, hepatic dysfunction, chronic kidney disease, proteinuria, and bleeding risk. The most common reason was poor hepatic function, with 48.6% of patients classified as Child–Pugh B or C. Most of these patients (91.4%) fell outside of the enrollment criteria for representative trials using combination immunotherapy. At the time of data analysis, nine patients had died while 14 were still undergoing monotherapy with durvalumab. Ten patients continued treatment for over 6 months. Of the 21 patients who discontinued treatment, disease progression was the primary reason for discontinuation (15 patients), while trAEs were the primary reason for discontinuation in 4 patients. Following disease progression, 5 patients were transitioned to alternative treatments, including one to regorafenib, one to cabozantinib, one to hepatic arterial infusion chemotherapy and two to lenvatinib. Six patients continued treatment for lacking alternative options. Two patients with stable disease transitioned to other therapies, despite the presence of controlled intrahepatic tumors. One patient crossed over to atezolizumab plus bevacizumab owing to a diminished risk of bleeding.

### 3.3 Effectiveness of Durvalumab Monotherapy

Median PFS was 2.7 months (95% CI: 1.774–6.177) but median OS was not reached (95% CI: 7.261–NA). Six‐month and 12‐month survival rates were 85.1% and 50.2%, respectively. Best overall responses included one partial response (PR) (2.9%) and 17 stable disease (SD) (48.6%). The ORR was 2.9%, and DCR was 51.4%. No significant correlation was found between imAEs and best overall response (*p* = 0.0528), though this analysis was limited by only four patients experiencing imAEs. Figure 1B displays the change (%) in tumor diameter from baseline to the best response. Cox regression multivariate analysis for OS considered factors such as age ≥ 80 years, Child–Pugh B or C, BCLC stage C, and alpha‐fetoprotein (AFP) > 400 ng/mL. No significant prognostic factors were identified in this study.

### 3.4 Safety of Durvalumab Monotherapy

Table S1 lists AEs noted during the observation period. The most frequent AEs were increased AST (34.3%), hypoalbuminemia (28.6%), increased ALT (25.7%), and increased bilirubin (17.1%). Grade ≥ 3 trAEs affected 8.6% of patients, including AST/ALT elevation, hypoalbuminemia, and diarrhea (one case each). Liver dysfunction (defined as encephalopathy, massive ascites, or jaundice [18]) occurred in 2 patients (5.7%). Four patients (11.4%) discontinued treatment due to AEs, including AST and ALT increased, diarrhea, tumor rupture, and deterioration of general condition. ImAEs were observed in five patients (14.3%), including two cases of grade ≥ 3 imAEs (hepatotoxicity and diarrhea), both occurring in patients who had been treated for over 6 months. High‐dose steroid therapy (prednisolone ≥ 1.0 mg/kg/day) was only used for the patient with grade 3 diarrhea due to immune‐related colitis. Our analysis of ALBI scores over a 12‐week period following durvalumab initiation revealed distinct patterns between groups. The mean ALBI scores were tracked at baseline and weeks 4, 8, and 12, as illustrated in Figure 1C,D. Mixed‐effects model analysis demonstrated significant liver function deterioration in the PD group (*p* = 0.016), while the non‐PD group maintained stable scores (*p* = 0.771) at the 12‐week mark (Figure 1C). Among Child–Pugh B or C patients exclusively, ALBI scores worsened significantly in the PD group (*p* = 0.012) but remained stable in the non‐PD group (*p* = 0.363), suggesting that liver function deterioration was linked to disease progression (Figure 1D). These findings suggest that disease progression, rather than durvalumab treatment, was the primary driver of liver function decline.

## 4 Discussion

This study examined the safety and effectiveness of durvalumab monotherapy in a real‐world cohort of advanced HCC patients ineligible for combination immunotherapy. Our findings suggest durvalumab monotherapy may be a viable option for these patients, addressing an unmet need in advanced HCC management.

The cohort included patients often excluded from trials, with nearly half having Child–Pugh B liver function and others with proteinuria and chronic kidney disease. Notably, 91.4% were ineligible for IMbrave150 or HIMALAYA, with the remainder aged 80 or older. This reflects global HCC demographic shifts, especially in countries like Japan, where older populations increasingly present with MASLD [19, 20, 21]. Despite complex profiles, the safety of durvalumab was favorable, with grade ≥ 3 trAEs in 8.6% and an 11.4% discontinuation rate, comparable to the HIMALAYA trial [2]. These findings align with the CheckMate 040 trial on nivolumab's safety in Child–Pugh B patients and a meta‐analysis of studies (699 Child–Pugh B, 2114 Child–Pugh A), which found similar safety profiles despite lower response rates in Child–Pugh B patients [14, 22]. Durvalumab monotherapy may thus offer a viable option for underrepresented groups, including those with Child–Pugh B liver function, renal issues, or bleeding risk. Our ORR, DCR, and median PFS are comparable to VEGF‐TKIs, particularly in Child–Pugh B patients [9, 10, 11]. Reports suggest VEGF‐TKIs are effective for tumors in Child–Pugh B cirrhosis but often cause significant AEs, highlighting the need for safer alternatives [9, 10, 11]. Challenges with lenvatinib in poor liver function and mixed real‐world data on atezolizumab plus bevacizumab in Child–Pugh B patients underscore the complexity of treating this population [23, 24]. Further studies are needed to identify the best treatments for Child–Pugh B HCC patients, especially those unable to use VEGF inhibitors due to bleeding risk.

In conclusion, durvalumab monotherapy shows promise for patients ineligible for combination immunotherapy, particularly those with Child–Pugh B status, renal dysfunction, and other common comorbidities in aging HCC populations. As HCC demographics evolve, prospective studies are needed to confirm these findings in challenging patient groups.

## Author Contributions

**Chihiro Miwa:** writing – original draft, investigation, data curation, visualization, validation, formal analysis. **Sadahisa Ogasawara:** conceptualization, methodology, supervision, project administration, writing – review and editing, data curation, investigation, validation, formal analysis, visualization. **Takuya Yonemoto:** data curation, investigation. **Sae Yumita:** data curation, investigation. **Tomomi Okubo:** data curation, investigation. **Miyuki Nakagawa:** data curation, investigation. **Keisuke Koroki:** data curation, investigation. **Masanori Inoue:** data curation, validation, formal analysis, visualization, writing – original draft, investigation. **Naoya Kanogawa:** investigation, data curation. **Masato Nakamura:** investigation, data curation. **Takayuki Kondo:** data curation, investigation. **Shingo Nakamoto:** data curation, investigation. **Norio Itokawa:** data curation, investigation. **Masanori Atsukawa:** data curation, investigation. **Ei Itobayashi:** data curation, investigation. **Naoya Kato:** supervision.

## Ethics Statement

All procedures followed the ethical standards of the institutional and/or national research committee and the 1964 Declaration of Helsinki, including its later amendments or equivalent ethical standards. This study was approved by the Research Ethics Committee of Chiba University (HK202309‐02). Written consent was not required for this type of study, in accordance with Japan's Ethical Guidelines for Medical and Biological Research Involving Human Subjects.

## Conflicts of Interest

Sadahisa Ogasawara received honoraria from Bayer (Leverkusen, Germany), Eisai (Tokyo, Japan), Eli Lilly (Indianapolis, IN, USA), Chugai Pharma (Tokyo, Japan), AstraZeneca (Cambridge, UK), and Merck & Co. Inc. (Kenilworth, NJ, USA); consulting or advisory fees from Bayer, Eisai, Merck & Co. Inc., Chugai Pharma, Eli Lilly, and AstraZeneca; and research grants from Bayer, AstraZeneca, and Eisai. Masanori Atsukawa has received a research grant from Eisai. Naoya Kato received honoraria from Bayer, Eisai, Sumitomo Dainippon Pharma (Tokyo, Japan), and Merck & Co. Inc.; consulting or advisory fees from Bayer and Eisai; and research grants from Bayer and Eisai. The remaining authors declare no conflicts of interest.

## Supporting information


**Table S1.** Summary of treatment‐related adverse event (trAE) and immune‐mediated adverse event (imAE).

## Data Availability

The data that support the findings of this study are available from the corresponding author upon reasonable request.

## References

[1] R. S. Finn , S. Qin , M. Ikeda , et al., “Atezolizumab Plus Bevacizumab in Unresectable Hepatocellular Carcinoma,” New England Journal of Medicine 382, no. 20 (2020): 1894–1905, 10.1056/NEJMoa1915745. 32402160

[2] G. K. Abou‐Alfa , G. Lau , M. Kudo , et al., “Tremelimumab Plus Durvalumab in Unresectable Hepatocellular Carcinoma,” NEJM Evidence 1, no. 8 (2022): EVIDoa2100070, 10.1056/EVIDoa2100070. 38319892

[3] P. R. Galle , T. Decaens , M. Kudo , et al., “Nivolumab (NIVO) Plus Ipilimumab (IPI) Vs Lenvatinib (LEN) or Sorafenib (SOR) as First‐Line Treatment for Unresectable Hepatocellular Carcinoma (uHCC): First Results From CheckMate 9DW,” Journal of Clinical Oncology 42, no. 17_suppl (2024): LBA4008, 10.1200/JCO.2024.42.17_suppl.LBA4008.

[4] The Japan Society of Hepatology , “Algorithm for Drug Therapy in Hepatocellular Carcinoma. JSH Clinical Practice Guidelines,” (2021), https://www.jsh.or.jp/lib/files/medical/guidelines/jsh_guidlines/medical/guideline_jp_2021_cq39_algorithm.pdf.

[5] A. Vogel , E. Martinelli , A. Cucchetti , et al., “Updated Treatment Recommendations for Hepatocellular Carcinoma (HCC) From the ESMO Clinical Practice Guidelines,” Annals of Oncology 32, no. 6 (2021): 801–805, 10.1016/j.annonc.2021.02.012. 33716105

[6] J. D. Gordan , E. B. Kennedy , G. K. Abou‐Alfa , et al., “Systemic Therapy for Advanced Hepatocellular Carcinoma: ASCO Guideline Update,” Journal of Clinical Oncology 42, no. 15 (2023): 1342–1358, 10.1200/JCO.23.02745. 38502889

[7] T. Yau , J. W. Park , R. S. Finn , et al., “Nivolumab Versus Sorafenib in Advanced Hepatocellular Carcinoma (CheckMate 459): A Randomised, Multicentre, Open‐Label, Phase 3 Trial,” Lancet Oncology 23, no. 1 (2022): 77–90, 10.1016/S1470-2045(21)00604-5. 34914889

[8] R. S. Finn , B.‐Y. Ryoo , P. Merle , et al., “Pembrolizumab as Second‐Line Therapy in Patients With Advanced Hepatocellular Carcinoma in KEYNOTE‐240: A Randomized, Double‐Blind, Phase III Trial,” Journal of Clinical Oncology 38, no. 3 (2020): 193–202, 10.1200/JCO.19.01307. 31790344

[9] S. Ogasawara , T. Chiba , Y. Ooka , et al., “Sorafenib Treatment in Child‐Pugh A and B Patients With Advanced Hepatocellular Carcinoma: Safety, Efficacy, and Prognostic Factors,” Investigational New Drugs 33, no. 3 (2015): 729–739, 10.1007/s10637-015-0225-1. 25861764

[10] K. Ogushi , M. Chuma , H. Uojima , et al., “Safety and Efficacy of Lenvatinib Treatment in Child‐Pugh A and B Patients With Unresectable Hepatocellular Carcinoma in Clinical Practice: A Multicenter Analysis,” Clinical and Experimental Gastroenterology 13 (2020): 385–396, 10.2147/CEG.S273694. 33061517 PMC7534867

[11] K. Kobayashi , S. Ogasawara , S. Maruta , et al., “A Prospective Study Exploring the Safety and Efficacy of Lenvatinib for Patients With Advanced Hepatocellular Carcinoma and High Tumor Burden: The LAUNCH Study,” Clinical Cancer Research 29, no. 23 (2023): 4760–4769, 10.1158/1078-0432.CCR-23-0830. 37796614

[12] A. Hiraoka , T. Tada , M. Hirooka , et al., “Efficacy of Durvalumab Plus Tremelimumab Treatment for Unresectable Hepatocellular Carcinoma in Immunotherapy Era Clinical Practice,” Hepatology Research (2024), 10.1111/hepr.14136. Epub ahead of print. 39526824

[13] S. Shimose , I. Saeki , T. Tomonari , et al., “Initial Clinical Experience With Durvalumab Plus Tremelimumab in Patients With Unresectable Hepatocellular Carcinoma in Real‐World Practice,” Oncology Letters 28, no. 2 (2024): 397, 10.3892/ol.2024.14530.eCollection2024Aug. 38979550 PMC11228928

[14] M. Kudo , A. Matilla , A. Santoro , et al., “CheckMate 040 Cohort 5: A Phase I/II Study of Nivolumab in Patients With Advanced Hepatocellular Carcinoma and Child‐Pugh B Cirrhosis,” Journal of Hepatology 75, no. 3 (2021): 600–609, 10.1016/j.jhep.2021.04.022. 34051329

[15] M. J. Ratain , T. Eisen , W. M. Stadler , et al., “Phase II Placebo‐Controlled Randomized Discontinuation Trial of Sorafenib in Patients With Metastatic Renal Cell Carcinoma,” Journal of Clinical Oncology 24, no. 16 (2006): 2505–2512, 10.1200/JCO.2005.03.6723. 16636341

[16] D. Semela and J. F. Dufour , “Angiogenesis and Hepatocellular Carcinoma,” Journal of Hepatology 41, no. 5 (2004): 864–880, 10.1016/j.jhep.2004.09.006. 15519663

[17] F. Gurevich and M. A. Perazella , “Renal Effects of Anti‐Angiogenesis Therapy: Update for the Internist,” American Journal of Medicine 122, no. 4 (2009): 322–328, 10.1016/j.amjmed.2008.11.025. 19332223

[18] S. Ogasawara , F. Kanai , S. Obi , et al., “Safety and Tolerance of Sorafenib in Japanese Patients With Advanced Hepatocellular Carcinoma,” Investigational New Drugs 29, no. 5 (2011): 850–856, 10.1007/s10637-010-9473-0. 21484134

[19] R. Tateishi , K. Uchino , N. Fujiwara , et al., “A Nationwide Survey on Non‐B, Non‐C Hepatocellular Carcinoma in Japan: 2011‐2015 Update,” Journal of Gastroenterology 54, no. 4 (2019): 367–376, 10.1007/s00535-019-01450-1. 30498904 PMC6437291

[20] H. Enomoto , Y. Ueno , Y. Hiasa , et al., “The Transition in the Etiologies of Hepatocellular Carcinoma‐Complicated Liver Cirrhosis in a Nationwide Survey of Japan,” Journal of Gastroenterology 56, no. 2 (2021): 158–167, 10.1007/s00535-020-01751-4. 33219410 PMC7862502

[21] R. Tateishi , T. Matsumura , T. Okanoue , et al., “Hepatocellular Carcinoma Development in Diabetic Patients: A Nationwide Survey in Japan,” Journal of Gastroenterology 56, no. 3 (2021): 261–273, 10.1007/s00535-020-01777-8. 33427937 PMC7932951

[22] E. Xie , Y. H. Yeo , B. Scheiner , et al., “Immune Checkpoint Inhibitors for Child‐Pugh Class B Advanced Hepatocellular Carcinoma: A Systematic Review and Meta‐Analysis,” JAMA Oncology 9, no. 10 (2023): 1423–1431, 10.1001/jamaoncol.2023.2605. 37615958 PMC10450588

[23] M. Rimini , M. Persano , T. Tada , et al., “Survival Outcomes From Atezolizumab Plus Bevacizumab Versus Lenvatinib in Child‐Pugh B Unresectable Hepatocellular Carcinoma Patients,” Journal of Cancer Research and Clinical Oncology 149, no. 10 (2023): 7565–7577, 10.1007/s00432-023-05143-7. 36976353 PMC11798051

[24] A. D'Alessio , C. A. M. Fulgenzi , N. Nishida , et al., “Preliminary Evidence of Safety and Tolerability of Atezolizumab Plus Bevacizumab in Patients With Hepatocellular Carcinoma and Child‐Pugh A and B Cirrhosis: A Real‐World Study,” Hepatology 76, no. 4 (2022): 1000–1012, 10.1002/hep.32630. 35313048 PMC9790703

