# Supplementary material for: Durvalumab Monotherapy in Complex Advanced Hepatocellular Carcinoma: A Real‐World Study of Patients Ineligible for Combination Immunotherapy
Source: Cancer Med. 2025 Feb 27;14(5):e70642. doi: 10.1002/cam4.70642 (PMC11866308; doi:10.1002/cam4.70642)
Supplement: Supplementary file 1 — Table S1. Summary of treatment‐related adverse event (trAE) and immune‐mediated adverse event (imAE). [file CAM4-14-e70642-s001.docx]

**Supplemental Table 1. Summary of treatment-related adverse event (trAE) and immune-mediated adverse event (imAE)**

|  | **trAE** | | **imAE** | |
| --- | --- | --- | --- | --- |
| **Variables** | **Any grade (%)** | **Grade ≥ 3 (%)** | **Any grade (%)** | **Grade ≥ 3 (%)** |
| AST increased | 34.3 | 2.9 | 5.7 | 2.9 |
| Hypoalbuminemia | 28.6 | 2.9 | 0 | 0 |
| ALT increased | 25.7 | 2.9 | 5.7 | 2.9 |
| Blood bilirubin increased | 17.1 | 0 | 0 | 0 |
| Ascites | 5.7 | 0 | 0 | 0 |
| Fatigue | 14.3 | 0 | 0 | 0 |
| Anorexia | 11.4 | 0 | 0 | 0 |
| Pruritus | 11.4 | 0 | 0 | 0 |
| Rash | 11.4 | 0 | 2.9 | 0 |
| Diarrhea | 8.6 | 2.9 | 5.7 | 2.9 |
| Insomnia | 8.6 | 0 | 0 | 0 |
| Proteinuria | 8.6 | 0 | 0 | 0 |
| Thyroid dysfunction | 5.7 | 0 | 2.9 | 0 |
| Liver dysfunction | 5.7 | - | - | - |

Liver dysfunction is defined as encephalopathy, massive ascites, or jaundice.

Abbreviations: AST, aspartate aminotransferase; ALT, alanine aminotransferase; trAE, treatment-related adverse event; imAE, immune-mediated adverse event
